# Isolation of a Mouse Motoneuron-Enriched Fraction from Mouse Spinal Cord on a Density Barrier

**DOI:** 10.1100/tsw.2002.841

**Published:** 2002-06-07

**Authors:** John Graham

**Affiliations:** School of Biomolecular Sciences, Liverpool John Moores University, UK

**Keywords:** motoneurons, mouse spinal cord, OptiPrep^TM^, iodixanol, density barrier

## Abstract

After a combined enzymic and mechanical disruption of the spinal cord tissue, the low-density motoneurons band at the interface of a 1.06-g/ml barrier through which other contaminating cells sediment.

**METHOD TYPE:** extraction, isolation, purification and separation

**SUB METHOD TYPE:** centrifugation

## INTRODUCTION

The growth and differentiation of spinal motoneurons are dependent on various genetic and epigenetic factors, which influence both functional and morphological characteristics[1]. Involvement of a number of trophic molecules is known to be an important part of these processes[1]. Consequently there is a considerable amount of research carried out on cultured motoneurons, which can be derived from embryonic mouse or rat spinal cord.

The general procedure can be summarized as follows: spinal cords are dissected from embryos and following a combined enzymic and mechanical disruption of the tissue, debris is removed by pelleting the cells through a cushion of bovine serum albumin. A motoneuron-rich fraction is then isolated from the cell pellet prior to cell culture.

Bataillé et al.[1] layered the crude cell pellet from rat embryo spinal cord over two layers of Nycodenz® of density, ρ = 1.047 and 1.065 g/ml and analyzed the cells that banded at the top of each Nycodenz® layer. The majority of the cells banded at the lower interface and the diameter of these cells (approx. 4.3 µm) was much lower than the minor population of cells (motoneurons) around the upper interface (approx. 6.7 µm). These large low-density cells contained very high levels of acetylcholine, which was virtually absent from the smaller denser cells at the lower interface.

It seems clear from this earlier method that the motoneurons, as a result of their larger size are less dense than the other cells. Duong et al.[2] have used a single layer of iodixanol ($\rho = 1.06$ g/ml) to purify mouse motoneurons, which remained on top of the density barrier. The following protocol is adapted from Ref. 2].

## MATERIALS AND EQUIPMENT

OptiPrep™ (60%, w/v iodixanol)
Hank's Balanced Salt Solution, without $Ca^{2+}$ and $Mg^{2+}$ (HBSS)
3.5% (w/v) Bovine serum albumin in HBSS (HBSS-BSA)
0.025% Trypsin in isotonic solution

Plastic conical centrifuge tubes (12–15 ml)
Plastic Pasteur pipette for overlayering
Low-speed (temperature-controlled) centrifuge with swinging-bucket rotor
Syringe with 21-gauge needle

## METHOD

1. Carry out all operations at room temperature.
2. After dissection of the mouse embryo spinal columns, incubate them in an isotonic 0.025% (w/v) trypsin solution for 20 min (see Note 1).
3. Dissociate the tissue by repeated passage through the syringe needle.
4. Layer the suspension over HBSS-BSA and centrifuge at 120*g* for 10 min to remove cell debris.
5. Discard the supernatant and resuspend the pellet in HBSS.
6. Dilute OptiPrep™ with HBSS to give a 1.06-g/ml solution, equivalent to 10.4% w/v iodixanol (see Notes 2 and 3).
7. Layer the resuspended pellet over the 1.06 g/ml solution.
8. Centrifuge at 400*g* for 25 min.
9. Collect the upper layer and centrifuge at 700*g* for 10 min to pellet the motoneuron fraction.
10. Wash the cell pellet as required. See Refs. [1,2] for information on motoneuron culture (see Note 4).

## NOTES

1. The detailed strategy for dissociation of the tissue will vary from laboratory to laboratory.
2. For motoneurons from other species it may be necessary to modulate the density of the lower layer. See Ref. [3] for more information about gradient solution preparation.
3. A four step discontinuous gradient system is described in Ref. [4] for the isolation of hippocampal neurons.

4. Duong et al.[2] identified motoneurons from E13-E14 Swiss mouse embryos by their expression of high levels of choline acyltransferase (ChAT). The authors were thus able to distinguish them from other cells, which express much less of the enzyme, using immunolabeling with an anti-ChAT antibody. The cultures contained at least 90% motoneurons based on this assessment.

## ACKNOWLEDGEMENTS

The author and TheScientificWorld wish to thank Axis-Shield PoC, AS, Oslo, Norway for their kind permission to adapt OptiPrep™ Application Sheet C22 in the preparation of this Protocol Article.
